# PLEKHS1 Over-Expression is Associated with Metastases and Poor Outcomes in Papillary Thyroid Carcinoma

**DOI:** 10.3390/cancers12082133

**Published:** 2020-07-31

**Authors:** Xiangling Xing, Ninni Mu, Xiaotian Yuan, Na Wang, C. Christofer Juhlin, Klas Strååt, Catharina Larsson, Dawei Xu

**Affiliations:** 1 Department of Medicine, Division of Hematology, Bioclinicum and Center for Molecular Medicine, Karolinska University Hospital Solna and Karolinska Institutet, SE-171 76 Stockholm, Sweden; Xiangling.Xing@ki.se (X.X.); Klas.Straat@ki.se (K.S.); Dawei.Xu@ki.se (D.X.); 2 Department of Oncology-Pathology, Karolinska Institutet, Karolinska University Hospital Solna, Bioclinicum, SE-171 76 Stockholm, Sweden; Ninni.Mu@ki.se (N.M.); na.wang@ki.se (N.W.); christofer.juhlin@ki.se (C.C.J.); 3 Department of Clinical Pathology and Cytology, Karolinska University Hospital, SE-171 76 Stockholm, Sweden

**Keywords:** metastasis, PLEKHS1, prognostic factors, promoter mutations, TERT, thyroid carcinoma

## Abstract

Pleckstrin homology domain containing S1 (PLEKHS1) is a poorly characterized factor, although its promoter mutations were identified in human malignancies including thyroid carcinoma (TC). This study was designed to determine *PLEKHS1* promoter hotspot mutations in papillary and anaplastic thyroid carcinomas (PTCs and ATCs) and to evaluate if *PLEKHS1* expression influences clinical outcome. The *PLEKHS1* promoter mutation was observed in 1/93 of PTCs and none of 18 ATCs in our cohort; however, *PLEKHS1* expression was aberrantly up-regulated in TCs compared to adjacent non-tumorous thyroid tissues. ATC tumors, an undifferentiated TC, exhibited the highest *PLEKHS1* expression. In both TCGA and present cohorts of PTCs, *PLEKHS1* gene methylation density was inversely correlated with its mRNA expression and demethylation at the *PLEKHS1* locus occurred at two CpGs. Higher *PLEKHS1* expression was associated with lymph node and distant metastases, and shorter overall and disease-free survival in our cohort of PTC patients. Importantly, *PLEKHS1* over-expression predicted shorter patient survival in PTCs lacking *TERT* promoter mutations. Cellular experiments showed that *PLEKHS1* over-expression enhanced AKT phosphorylation and invasiveness. Collectively, the *PLEKHS1* gene demethylation causes its over-expression in PTCs. *PLEKHS1* promotes aggressive behavior of TCs possibly by increasing AKT activity, and its over-expression predicts poor patient outcomes.

## 1. Introduction

Papillary thyroid carcinoma (PTC), the most common type of thyroid cancer (TC) originating from follicular thyroid cells, accounts for more than 80% of all thyroid malignancies [1,2]. The vast majority of PTCs exhibit an indolent nature with favorable outcomes, but 10–15% of localized and R0 resected PTCs will eventually relapse or metastasize, and develop treatment resistance, thereby leading to disease-related morbidity/mortality [1,2,3,4]. Thus, it is clinically important to precisely stratify these aggressive PTCs for timely intervention and close surveillance. A number of clinical variables including age, tumor size, extrathyroidal extension, lymph node status, and the occurrence of distant metastasis are routinely applied to correctly stage TCs in general. However, despite their usefulness in choosing therapeutic approaches, these factors remain insufficient to predict PTC progression and recurrence post-surgical treatment. Anaplastic thyroid carcinoma (ATC) is an undifferentiated form of TC that might originate from pre-existing well-differentiated thyroid carcinomas including PTC or occur de novo [1,2].

Recent advances in high-throughput next-generation sequencing technologies have led to a revolution in cancer genomics including PTCs and ATCs, providing deep insights into the molecular pathogenesis and facilitating the identification of new biomarkers for the disease prognostication [2,3,5,6,7]. Recurrent promoter mutations (C228T/C250T) of the *telomerase reverse transcriptase (TERT)* gene were recently identified in PTCs and ATCs, and the presence of this genetic event predicts poor patient outcomes [8,9,10,11,12,13,14]. Moreover, the *TERT* promoter mutation activates *TERT* gene transcription [8,15], and higher *TERT* expression is similarly associated with aggressive PTCs [9,11,14,16].

**Figure 1:**
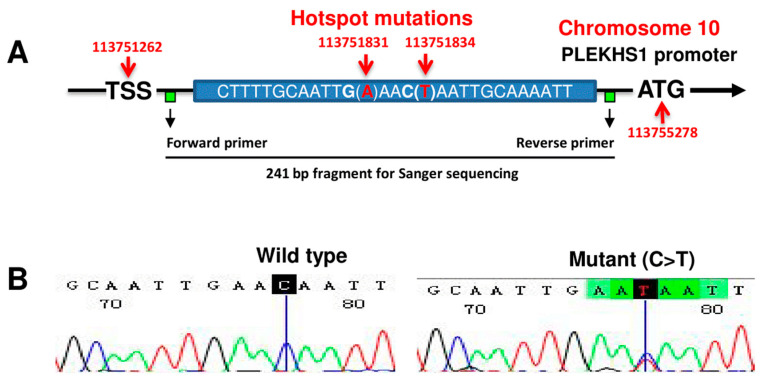
*PLEKHS1* promoter mutations are rare in PTCs. (**A**) Schematics of hotspot mutations in the *PLEKHS1* promoter (based on the GRCh38.p13). Two mutation hotspots on chromosome 10 are indicated (G > A 113751831) and (C > T 113751834), respectively. Of note, these two mutations are flanked by stretches of 10 bp on both sides that are palindromic to each other. TSS: Transcription start site. The locations of primers for Sanger sequencing are indicated. (**B**) Wild-type (wt) and mutant *PLEKHS1* promoter sequences as determined using Sanger sequencing in PTC tumors. A representative chromatogram of a PTC tumor with a wt *PLEKHS1* promoter is shown in the left panel. Shown in the right panel is the only PTC tumor carrying the *PLEKHS1* promoter mutation.

Although *TERT* promoter mutations are very helpful for PTC prognostication, they occur only in a small fraction (5–25%) of patients [8,14,16], and it remains challenging to accurately foresee the outcome of patients carrying a wild-type (wt) *TERT* promoter. In search for new prognostic factors, the *Pleckstrin homology domain containing S1* (*PLEKHS1*) gene has attracted our attention, because hotspot *PLEKHS1* promoter mutations (Figure 1A) have also been shown to occur in different types of human cancer including TC and other malignancies where *TERT* promoter mutations are frequently observed [17,18]. In the present study, we analyzed the *PLEKHS1* gene for its promoter mutation and expression. Our results show that the *PLEKHS1* hotspot promoter mutations are rare, but its higher expression is significantly associated with metastasis and shorter patient survival in PTCs.

## 2. Results

### 2.1. The Rarity of PLEKHS1 Hotspot Promoter Mutations in PTCs and ATCs

A hotspot *PLEKHS1* promoter mutation was shown to occur in one PTC out of nine analyzed TCs, in an earlier report [17] (Figure 1A), which suggests that this genetic event might be recurrent in PTC. We thus analyzed the *PLEKHS1* promoter region for the hotspot mutations in five TC cell lines, primary PTC tumors from 93 patients and ATCs from 18 patients using Sanger sequencing. The sequencing analyses revealed that all five cell lines were wild-type, while only one PTC tumor was mutation-positive (Figure 1B). This was derived from a 75 years old female patient that in addition to the *PLEKHS1* promoter-hotspot mutation also carried a *TERT* promoter C250T mutation but lacked the *BRAF*^V600E^ mutation. She had lymph node and distant metastases, and relapsed or progressed in her disease 6 months after surgery. These results clearly demonstrate the rarity of the *PLEKHS1* promoter mutation in PTCs and ATCs.

### 2.2. PLEKHS1 Expression in TC Cell Lines and Primary PTC and ATC Tumors

**Figure 2:**
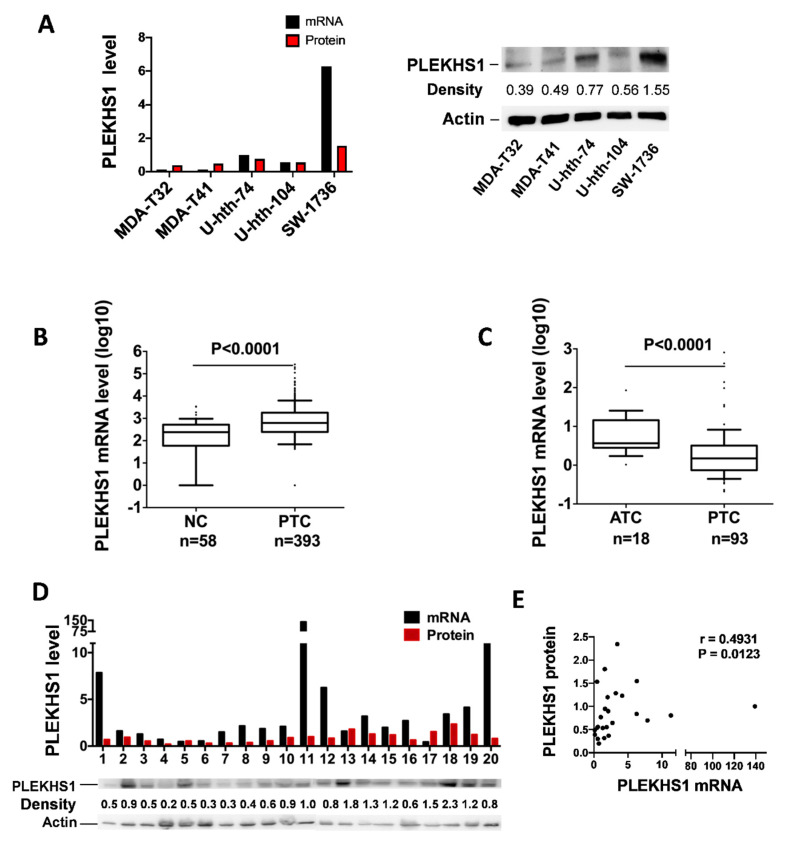
PLEKHS1 is over-expressed in thyroid cancer (TC)-derived cell lines and primary tumors. (**A**) PLEKHS1 expression in TC cell lines. Left and right: PLEKHS1 mRNA and protein expression levels as determined using qPCR and immunoblotting, respectively. The quantification results (band intensity) in the right panel are shown in the left panel. (**B**) Differential expression of *PLEKHS1* mRNA between non-cancerous adjacent thyroid tissues and primary PTC tumors. The TCGA cohort of PTC tumors and adjacent tissues are analyzed and mRNA levels were expressed as FPKM (Fragments Per Kilobase Million). (**C**) Higher levels of *PLEKHS1* expression in ATC tumors than PTCs. (**D**) PLEKHS1 mRNA and protein expression levels in 20 PTC tumors as determined using qPCR and immunoblotting, respectively. The quantification results (band intensity) in the bottom panel are shown in the top panel. (**E**) Positive correlation between *PLEKHS1* mRNA and protein expression, as revealed by the analysis of combined results from both TC cell lines (**A**) and primary PTC tumors (**D**).

We then determined *PLEKHS1* expression in five cell lines and 111 primary tumors (93 PTCs and 18 ATCs). *PLEKHS1* mRNA was detected in all three ATC-derived cell lines with the highest expression in SW1736 cells. Interestingly, PTC-derived MDA-T32 and MDA-T41 cells expressed the lowest levels of *PLEKHS1* mRNA (Figure 2A), and similar findings were obtained for *PLEKHS1* protein levels in these cells (Figure 2A), indicating transcriptional control of this gene. We then analyzed *PLEKHS1* expression in primary tumors and non-cancerous thyroid tissues. In the TCGA dataset, comparison between PTCs and non-cancerous thyroid tissue samples showed a significant increase in *PLEKHS1* mRNA expression in tumors (239.8 (0–3445) vs. 624.9 (0–263865) median (Min–Max), *p* < 0.0001) (Figure 2B). For PTCs and ATCs, their *PLEKHS1* mRNA quantifications were 1.5 (0.2–811.9) and 3.7 (1.0–85.6), (median (Min–Max)), respectively, in our cohort (*p* < 0.0001, Figure 2C). Thus, ATC tumors expressed significantly higher levels of *PLEKHS1* mRNA than did PTCs. In addition, 20 of 93 PTC tumors were also analyzed for their *PLEKHS1* protein expression using immunoblotting assays. Their mRNA and protein levels of *PLEKHS1* were in general consistent with each other, although variations occurred to certain extents and correlation was not statistically significant (Figure 2D, *r* = 0.338, *p* = 0.145). However, the analysis of combining both 5 TC cell lines and 20 primary PTC tumors revealed a significantly positive correlation between *PLEKHS1* mRNA and protein (Figure 2E, *r* = 0.493, *p* = 0.0123). Taken together, these results obtained from cell lines and primary tumors suggest that *PLEKHS1* mRNA over-expression aberrantly occurs in PTCs and ATCs.

### 2.3. Association Between PLEKHS1 Over-Expression and Metastases and Shorter Survival in PTCs

**Figure 3:**
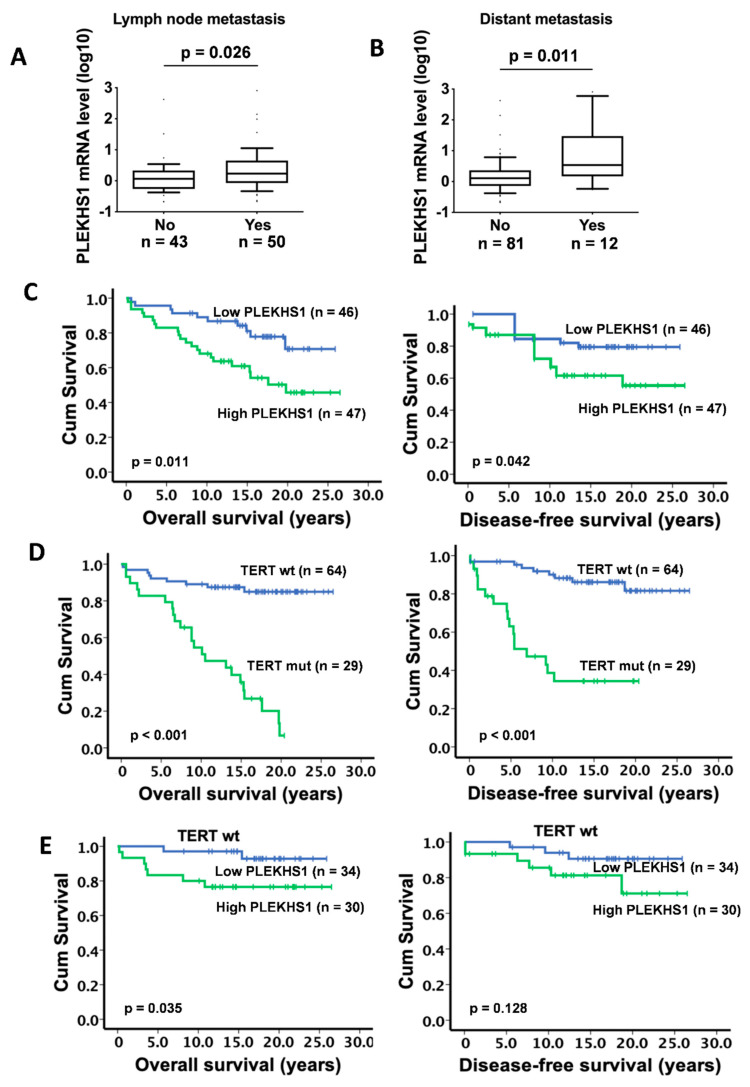
Higher *PLEKHS1* expression is associated with metastasis and shorter survival in PTC patients. (**A**,**B**) Higher *PLEKHS1* expression is associated with both lymph node and distant metastases. (**C**) Higher *PLEKHS1* expression predicts significantly shortened overall and disease-free survival (OS & DFS) time in PTC patients. (**D**) The presence of *TERT* promoter mutation is associated with significantly shorter OS and DFS in PTC patients. (**E**) The expression level of *PLEKHS1* is associated with OS but not DFS in PTC patients with wt *TERT* promoter.

Given the findings presented above, we sought to determine whether *PLEKHS1* is associated with PTC progression and/or patient prognosis. Lymph node metastasis occurred in 50/93 of PTC patients and the primary tumors from these patients expressed significantly higher levels of *PLEKHS1* mRNA than those without lymph node metastasis (*p* = 0.026) (Figure 3A and Table 1). Similarly, *PLEKHS1* expression was significantly higher in tumors from patients having distant metastases (12 cases) than those (81 cases) lacking metastases (*p* = 0.011) (Figure 3B and Table 1). The relationship between *PLEKHS1* expression and patient survival was further evaluated. The median expression level in tumors was used as a cut-off to divide the patients into low- and high-expressing groups. As shown in Figure 3C, the low-expressing group had significantly longer overall survival (OS) and disease-free survival (DFS) (*p* = 0.011 and 0.042, respectively). When *PLEKHS1* mRNA expression was used as a continuous variable, *p* values for OS and DFS were <0.001 and 0.004, respectively (Table 1). *PLEKHS1* expression was not associated with patient age, gender, tumor size, or extrathyroidal extension (Table 1). We also analyzed the relationship between *PLEKHS1* expression and clinico-pathological variables or *TERT* promoter statuses in 18 patients with ATC. As summarized in Table 2, there was no association of *PLEKHS1* expression with age, gender, tumor size, survival, and *TERT* promoter mutations or *TERT* expression.

It is well known that *TERT* promoter mutations are associated with shorter patient survival in PTCs [9,11], however, the majority of these patients carry a wt promoter and it is thus clinically important to discriminate between high and low risk patients in this category. To test whether *PLEKHS1* can serve as such a marker, we determined its association with *TERT* hotspot promoter mutations and patient survival. As demonstrated previously, the presence of *TERT* promoter mutations predicted substantially shorter OS and DFS in PTC patients (Figure 3D). A comparison of *PLEKHS1* expression between tumors that were wt and mutant for *TERT* hotspot promoter mutations did not show a difference, and moreover, *PLEKHS1* expression was not associated with survival in cases with *TERT* promoter mutation (*p* = 0.734 and 0.621 for OS and DFS, respectively). However, in the group with a wt *TERT* promoter, higher *PLEKHS1* expression was significantly associated with shorter OS (*p* = 0.035) and showed a tendency toward shorter DFS, although not reaching statistical significance (*p* = 0.128) (Figure 3E).

In multivariate analyses, *PLEKHS1* over-expression, age (≥55 years old) and larger tumor size (>4 cm), but not gender and *TERT* promoter mutations, were independently associated with shorter patient OS (Table S1). However, *PLEKHS1* over-expression and *TERT* promoter mutations had no effects on DFS, whereas tumor size remained as a predictor for poor DFS (Table S2).

We also analyzed the TCGA cohort of 393 patients with PTC for the relationship between *PLEKHS1* expression and clinico-pathological variables. There was a significant association between *PLEKHS1* over-expression and lymph node metastasis (*p* = 0.002) (Table S3), but not distant metastasis, likely due to the very few patients with distant metastasis (4/249) in the TCGA cohort. *PLEKHS1* expression levels were not associated with patient survival (Table S3), whereas the presence of *TERT* promoter mutations predicted significantly shorter OS and DFS, as shown previously [9]. In PTCs with a wt *TERT* promoter, *PLEKHS1* expression had no effects on patient survival, likely due to the low number of deaths during the follow-up period (7/281).

### 2.4. Enhanced AKT Phosphorylation, Proliferation and Invasiveness Mediated by PLEKHS1 Over-Expression in TC Cells

**Figure 4:**
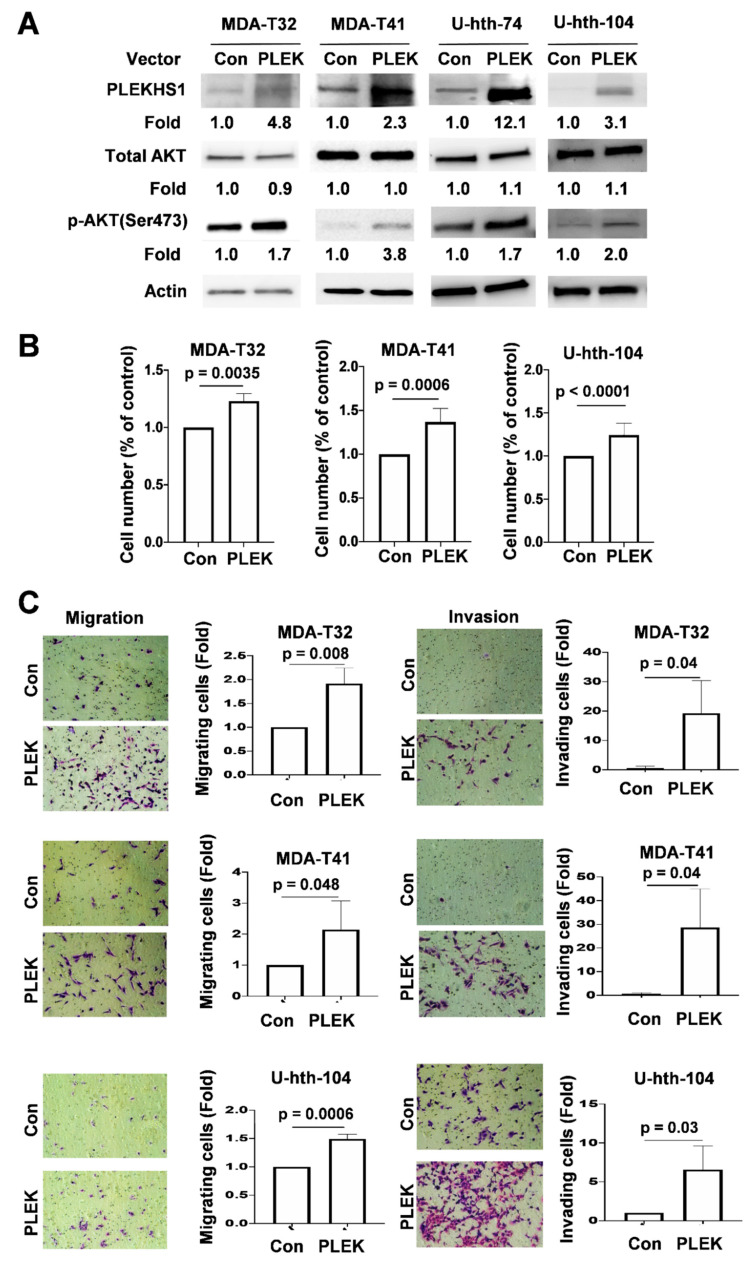
PLEKHS1 over-expression promotes AKT phosphorylation, proliferation and migration/invasion in PTC- and ATC-derived cells. (**A**) Increased AKT phosphorylation in PLEKHS1-transfected cells. MDA-T32, MDA-T41, U-hth-74, and U-hth-104 cells were transfected with control (Con) empty and PLEKHS1 (PLEK) expression vectors, respectively, and harvested 48 h post-transfection for total AKT and phosphorylated AKT levels using immunoblotting. One representative experiment out of three independent experiments is shown. The fold change for PLEKHS1, total and phosphorylated AKT is indicated (based on their signal intensity normalized to β-actin). (**B**) Increased cell proliferation of cells overexpressing PLEKHS1. MDA-T32, MDA-T41 and U-hth-104 cells above were incubated for 48 h and then counted. The number of control cells was set as 100%. Shown are the results from three independent experiments. (**C**) Enhanced migration and invasion of PLEKHS1-overexpressed cells. MDA-T32, MDA-T41 and U-hth-104 cells above were analyzed for their migration and invasion capacities using a transwell assay system. Left: representative images of migration or invasion. Right: the quantification of migrating or invading cells based on three independent experiments.

Because pleckstrin homology domains (PH domains) contained in PLEKHS1 bind phosphoinositides, and PLEKHS1 interacts with PI3KR3 [19], we hypothesize that PLEKHS1 may be associated with the activation of the PI3K-AKT pathway. To explore this possibility, we over-expressed PLEKHS1 in TC cells (two PTC-derived and two ATC-derived cell lines) by ectopically introducing its expression vector, and determined the effect on AKT phosphorylation as an indicator of activation of the PI3K/AKT pathway. As shown in Figure 4A, the ectopic expression of PLEKHS1 substantially enhanced the level of phosphorylated AKT, but did not affect the total AKT expression significantly, in both PTC and ATC cells.

The phosphorylated AKT exhibits an increased oncogenic activity, promoting cancer cell proliferation, survival and invasiveness or metastasis. Thus, we further determined alterations in proliferation and migration/invasion of PLEKHS1-overexpressed MDA-T32, MDA-T41 and U-hth-104 cells. As shown in Figure 4B, moderate, but significantly increased cell numbers were observed in PLEKHS1-transfected cells. Of note, these PLEKHS1-overexpressed cells acquired highly enhanced capacities in migration and invasion (Figure 4C). Especially for MDA-T32 and MDA-T41 cells, PLEKHS1 overexpression led to robust increases of invading cells (MDA-T32: 29-fold increase and MDA-T41: 43-fold increase) (Figure 4C). These results are highly consistent with PLEKHS1 overexpression in metastatic PTC patients.

### 2.5. Association Between Demethylation and mRNA Expression of the PLEKHS1 Gene in PTCs

**Figure 5:**
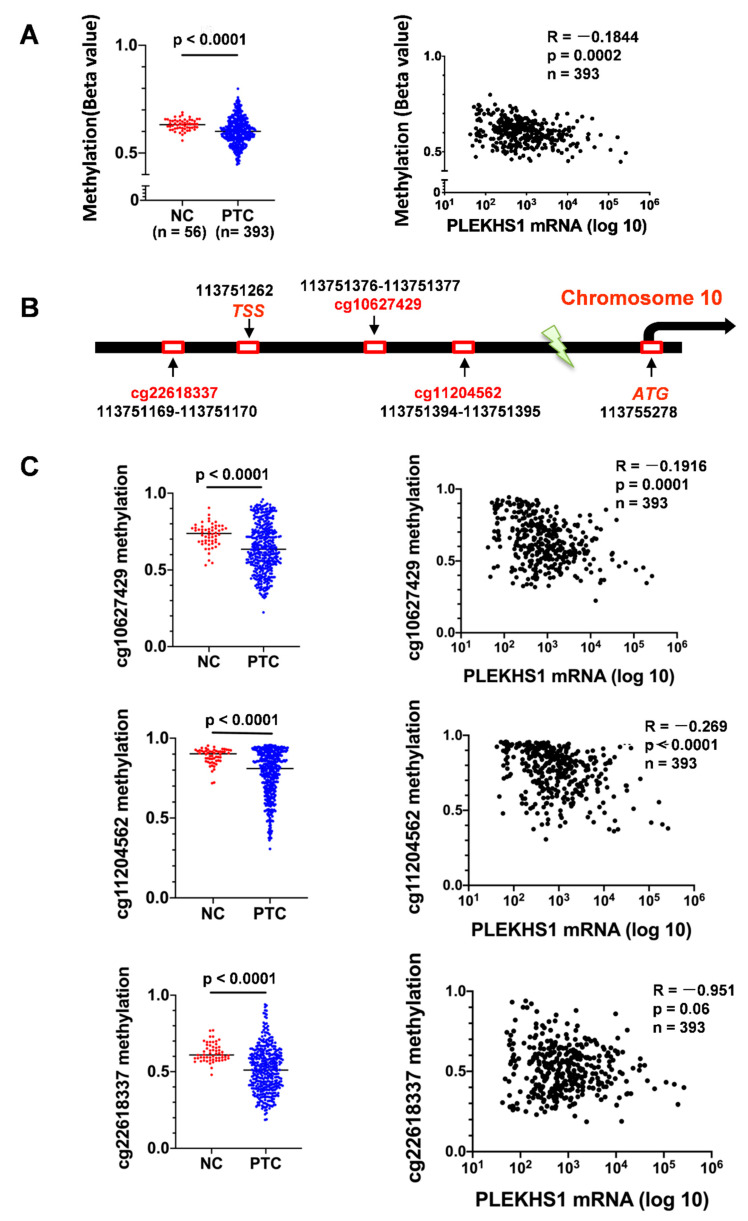
DNA methylation at the *PLEKHS1* locus is inversely correlated with *PLEKHS1* mRNA expression in the TCGA cohort of PTC patients. The analyses were performed on the DNA methylation at the *PLEKHS1* locus from the TCGA cohort of PTC patients (393 tumors and 56 adjacent non-tumorous thyroid tissues) (downloaded from The Cancer Genome Atlas (TCGA) [20]. The level of DNA methylation was arbitrarily expressed as β values (defined as the ratio of the signal intensity between methylated and unmethylated CpGs). *PLEKHS1* mRNA levels were expressed as FPKM (Fragments Per Kilobase Million). (**A**) Left panel: Significantly reduced *PLEKHS1* gene methylation in PTC tumors compared to their adjacent non-tumorous tissues. Right panel: Negative correlation between *PLEKHS1* methylation and mRNA expression in PTC tumors. (**B**) The locations of 3 CpG sites with differential methylation at the *PLEKHS1* locus between PTC tumors and non-tumorous tissues. TSS: Transcription start site. (**C**) The differential methylation levels at three specific CpG sites between PTC tumors and non-tumorous tissues and their negative correlation with *PLEKHS1* mRNA expression in PTC tumors.

Because the *PLEKHS1* promoter mutation is rare in PTCs as described above, we sought to determine whether *PLEKHS1* over-expression results from altered DNA methylation in tumors. To explore this, we first analyzed the methylation density at the *PLEKHS1* locus in the TCGA database. The methylation data are available for 393 PTC tumors and 56 adjacent normal thyroid tissue samples. As shown in Figure 5A (Left panel), the overall methylation level was significantly lower in PTC tumors than their adjacent non-tumorous tissues (*p* < 0.0001). Moreover, a significantly inverse correlation between the methylation and mRNA expression of the *PLEKHS1* gene was observed (*R* = −0.184, *p* = 0.0002) (Figure 5A, right panel). Furthermore, we specifically identified differentially methylated CpGs at the *PLEKHS1* locus (Figure 5B), which include cg10627429 (*p* < 0.0001), cg11204562 (−*p* < 0.0001), and cg22618337 (−*p* < 0.0001) (Figure 5C). In all these three CpGs, the methylation level was inversely correlated with *PLEKHS1* mRNA levels in PTC tumors (*R* = −0.192, −0.268 and −0.095, respectively, *p* < 0.0001, < 0.0001 and = 0.06, respectively) (Figure 5C), although the significance for cg22618337 is at a borderline level. Of note, all three CpGs are localized in the 5′ regulatory region of the *PLEKHS1* gene (Figure 5B).

**Figure 6:**
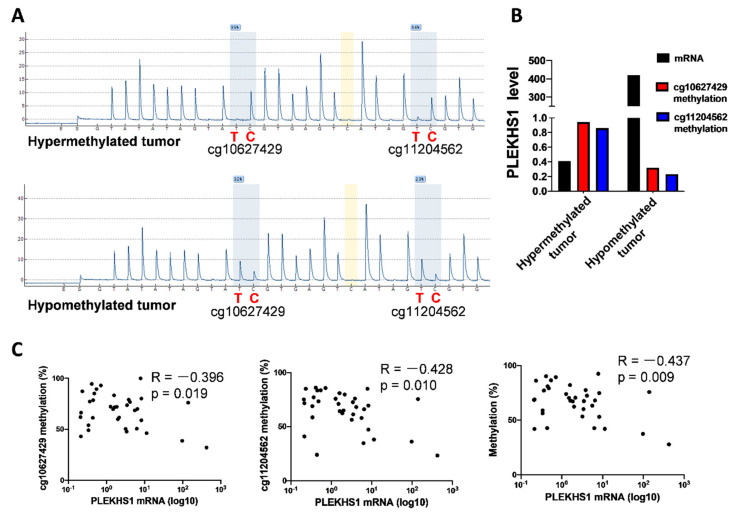
DNA methylation at the *PLEKHS1* promoter is inversely correlated with *PLEKHS1* mRNA expression in PTC patients. Pyrosequencing was performed to analyze the methylation status of cg10627429 and cg11204562 at the PLEKHS1 promoter in tumor DNA derived from 35 PTC patients, and the level of methylation at each CpG was expressed as percentage based on the PyroMark Q96 calculation. (**A**) The representative Pyrosequencing results from one patient with hypermethylation of both CpGs (Top) and one with hypomethylation (bottom). C (red): the methylated cytosine that is resistant to bisulfite conversion; T (red): the unmethylated cytosine that is conversed into uracil by bisulfite treatment. (**B**) *PLEKHS1* mRNA levels and methylation quantification (based on Figure 6A) in these two tumors. (**C**) The inverse correlation between the methylation of cg10627429 and/or cg11204562 and *PLEKHS1* expression in 35 PTC tumors.

To see if this is the case in our PTC cohort, we further determined the methylation status of cg10627429 and cg11204562, two key CpGs associated with PLEKHS1 expression as described above. Pyrosequencing was performed on tumor DNA derived from 35 PTC patients and we observed differential methylation levels at cg10627429 and cg11204562 in these 35 tumors. Figure 6A shows the methylation of cg10627429 and cg11204562 in two patients, one with hypermethylation (96% and 87%) while the other with hypomethylation of two CpGs. The hypomethylated tumor expressed more than 1000-fold higher *PLEKHS1* mRNA than did the hypermethylated one (422 vs. 0.41) (Figure 6B). Moreover, the analyses of 35 tumors reveal significantly inverse correlations between *PLEKHS1* expression and methylation status of each CpG (cg10627429 and cg11204562, *r* = −0.396, and −0.428, *p* = 0.019 and 0.010, respectively) or their combination (*r* = −0.437, *p* = 0.009) (Figure 6C).

## 3. Discussion

The *PLEKHS1* gene encodes a PH domain-containing protein [17,21] _ENREF_14. It was previously shown that PLEKHS1 participated in blood glucose regulation and insulin resistance in obese rats, while its exact roles in physiological and pathological settings are currently unclear [21]. Nevertheless, the link of *PLEKHS1* to human cancer has recently been established due to the identification of mutations in the *PLEKHS1* promoter in several human malignancies including bladder, breast, lung, and thyroid cancer, as well as acute lymphocytic leukemia [17]. These mutations are single-nucleotide substitutions in the PLEKHS1 proximal promoter, among which two are hotspot mutated sites 50 bps upstream from the first intron, predominately C > T or G > A transitions [17]. Approximately 40% of urothelial bladder tumors carry *PLEKHS1* promoter mutations, exhibiting the highest frequency in all analyzed cancer types in TCGA datasets [17]. More interestingly, Weinhold et al. observed that the *PLEKHS1* promoter mutation was present in 1/9 of thyroid tumors [17], with an occurrence rate largely similar to that of *TERT* promoter mutations in PTCs [16]. However, we screened 111 primary PTC and ATC tumors and found the *PLEKHS1* promoter mutation in a single PTC sample. All five TC-derived cell lines harbor a wt promoter. Our results thus indicate that the *PLEKHS1* promoter mutation is a rare genetic event in PTCs and ATCs.

The key finding in the present study is the association between *PLEKHS1* expression and PTC outcomes. Higher levels of *PLEKHS1* mRNA are associated with both lymph node and distant metastases and predict shorter OS and DFS in PTC patients. Moreover, *PLEKHS1* expression still serves as a prognostic factor for poor outcomes in PTCs with a wt *TERT* promoter. Because the vast majority of PTCs lack *TERT* promoter mutations and reliable prognostic factors for these patients are urgently needed, our present observations might be clinically useful. As of this, future applications of our initial observations, such as PLEKHS1 immunohistochemical analyses in PTCs, could merit future attention as potential clinical discriminators of poor-prognosis cases when the *TERT* promoter mutational screening is negative.

Consistent with the present results, Pignot et al. recently observed that PLEKHS1 was more frequently over-expressed in muscle-invasive bladder cancer (MIBC), an aggressive subtype of the disease, and its over-expression independently predicted shorter progression-free survival [18]. Moreover, PLEKHS1 over-expression promoted non-muscle invasive bladder cancer (NMIBC) progression to aggressive MIBC [18]. It is thus evident from these observations that PLEKHS1 plays an oncogenic role in the pathogenesis of PTC and bladder cancer. Even so, little is known about the functional activity of PLEKHS1. Pleckstrin homology domains (PH domains) can bind phosphoinositides, and are known for different ligand binding sites and for specificity of different ligands, thereby triggering intracellular signaling. Because PLEKHS1 interacts with PI3KR3 [19], while the AKT signaling is required for the PTC pathogenesis [22], we sought to determine its effect on the PI3K-AKT signaling. Indeed, the ectopic expression of PLEKHS1 in thyroid cancer cells increased the abundance of phosphorylated AKT. We further show that PLEKHS1 overexpression promotes proliferation and invasiveness of both PTC- and ATC-derived cells. Especially, PTC cell lines overexpressing PLEKHS1 exhibited a dramatic increase in their invasive capacity. This observation suggests the involvement of PI3K-AKT in PLEKHS1-mediated PTC progression. Further studies are required to elucidate how exactly PLEKHS1 enhances AKT phosphorylation and thereby contributes to aggressive PTCs.

It is currently unclear how PLEKHS1 expression is regulated. Interestingly, *PLEKHS1* mRNA abundance in the mutant *PLEKHS1* promoter-bearing PTC tumor was the fourth highest among all PTCs. However, the *PLEKHS1* promoter mutation occurs in almost the half of bladder tumors, but the presence of the mutation was not correlated with its mRNA expression [18], indicating a negligible effect of this genetic event on the *PLEKHS1* transcription. However, Weinhold et al. showed that the mutant *PLEKHS1* promoter-bearing tumors expressed lower levels of *PLEKHS1* mRNA according to their results obtained from the TCGA datasets [17]. Given these inconsistent observations, further molecular and biochemical dissections are apparently required to ascertain how exactly this mutational event influences the *PLEKHS1* gene transcription and contributes to oncogenesis. Based on the analysis of the TCGA cohort of PTC, we observed differential levels of DNA methylation at the *PLEKHS1* locus between normal thyroid tissues and PTC tumors, and moreover, a significantly negative correlation between *PLEKHS1* gene methylation and its mRNA expression was present in PTC tumors. Similarly, such an inverse correlation was observed in urothelial bladder tumors, too (Spearman correlation = −0.45, *p* = 8.34 × 10^−22^) [23,24]. Interestingly, all three CpGs are located in the *PLEKHS1* promoter region. By determining the methylation status of these CpGs in the present cohort of PTC tumors, we further demonstrate an inverse association of the methylation with *PLEKHS1* mRNA abundance. Thus, these results collectively suggest that the demethylation of the *PLEKHS1* promoter induces an open chromatin locally, thereby contributing to *PLEKHS1* over-expression during the pathogenesis of PTCs and bladder cancer. Further studies are required to elucidate the mechanisms underlying demethylation at the *PLEKHS1* locus during oncogenesis.

In addition to the hotspot mutation in the *PLEKHS1* promoter region as described above, its mutations in encoding sequences are also identified in bladder cancer and other malignancies [17,23,25]; and these mutations can be missense, truncating or inframe shifting. It remains unclear about the functional consequences of these different mutations, which calls for further investigations. Nevertheless, none of 399 PTC tumors were observed to bear mutations in the *PLEKHS1* encoding region, as determined using exon sequencing analyses [5]. This finding, together with our present results, suggests that the aberrant DNA demethylation rather than its sequence alteration is targeted for the pathogenesis of thyroid carcinomas.

## 4. Patients and Methods

### 4.1. Patients

The study includes 93 patients with PTC and 18 patients with ATC, previously described and characterized by Yuan et al. [9]. Ethical permission was obtained from the Regional Ethics Committee (Dnr 2015/959-31 on 24-07-2015) in Stockholm, and informed consent was given prior to sample collection. The PTC and ATC diagnoses were made by histopathological examination according to the 2004 World Health Organization (WHO) classification [26]. We excluded the patients diagnosed as follicular variant of PTC to avoid potential inclusion of non-invasive follicular thyroid neoplasms with papillary-like nuclear features according to the more recent WHO classification from 2017 [1]. PTC patients were operated 1987–2005 and ATC patients 1989–2007. Tumor samples were collected post-operatively and kept frozen at −80 °C until use following an established procedure where sample quality has been demonstrated over time [27]. After the exclusion of follicular variant of PTC, PTC samples were collected from the biobank and reviewed by a pathologist to confirm the representation of tumor cells in the frozen specimen. Clinical data, including age at diagnosis, gender, tumor size, lymph node, and distant metastasis status, was then retrospectively collected by reviewing the patient charts for the PTC cohort. Of the 12 patients with distant metastasis, two cases exhibited distant metastasis at diagnosis while the rest of the patients developed distant metastasis during the follow-up period. Follow-up data on OS (endpoints: dead or alive) and DFS (endpoints: relapsed/progression or disease-free) was also documented for PTC patients. Relapsed/progression was defined as local recurrence or metastasis during follow-up and disease-free was defined as no evidence of disease during the follow-up time after surgery. The last follow-up by reviewing patient charts was performed in September 2017. The detailed clinical information, selection process and frequency of TERT promoter mutations are reported and commented on as published [9]. Patient age, gender, lymph node status, and occurrence of distant metastases in relation to PLEKHS1 expression are summarized in Table 1 and 2 for PTC and ATC, respectively. Information on extrathyroidal extension (extension of tumor tissue into perithyroidal tissues including fat tissue according to AJCC 7th edition) was available for 81/93 PTC patients (Table 1) and TCGA cohort of PTCs.

The clinico-pathological data and *TERT* promoter status in the TCGA cohort of PTCs were downloaded via cBioPortal for Cancer Genomics [24,28,29], and PLEKHS1 mRNA abundances were arbitrarily expressed as RSEM (RNA-Seq by Expectation Maximization). The data for expression and methylation of the *PLEKHS1* gene in PTC tumors and adjacent non-tumorous tissues were directly downloaded from The Cancer Genome Atlas (TCGA) [20] The levels of *PLEKHS1* mRNA and DNA methylation were arbitrarily expressed as FPKM (Fragments Per Kilobase Million) and β values (the ratio of signal intensity between methylated and unmethylated CpGs), respectively.

### 4.2. Cell Lines and Cell Culture

The study included five TC-derived cell lines (MDA-T32, MDA-T41, U-hth-74, U-hth-104, and SW1736). ATC-derived cell lines U-hth-74, U-hth-104 and SW1736 were obtained from Dr. N-E Heldin and cytogenetically characterized [30]. Short tandem repeats (STR) genotyping for ATC-derived cell lines U-hth-74, U-hth-104 and SW1736 was recently performed and matched to previously published genotypes [9], while the PTC-derived cell lines MDA-T32 and MDA-T41 were purchased from ATCC in 2018. Cells were cultured in RPMI-1640 medium (Thermo Fisher Scientific, Waltham, MA) supplemented with 10% fetal bovine serum (Thermo Fisher Scientific, Waltham, MA), 100 U/mL penicillin, 100 μg/mL streptomycin, and 4 mM L-glutamine.

### 4.3. DNA Extraction and Sanger Sequencing

Genomic DNA was extracted from PTC tissues and cell lines using DNeasy Blood & Tissue Kit (Qiagen, Hilden, Germany) and the AllPrep Mini Kit (Qiagen) according to the manufacturer protocols, respectively. The mutational hotspots in *TERT* promoter region and the *BRAF* V600E mutational status was analyzed as described previously [9,11]. Sanger sequencing was applied for analyses of the *PLEKHS1* promoter mutation and performed at the KIGene core facility, Stockholm, Sweden, using the following primers: 5′-GAA TCC TCG GGA CAA GGC ACT-3′ (Forward) and 5′-GTC AGT CCT ATT TCC CTC TGA CT-3′ (Reverse). PCR was run for 40 cycles with an annealing temperature at 60 °C, which generated PCR products of 241 bp (Figure 1A). Sequencing data were analyzed by visual inspection of chromatograms and manual examination.

### 4.4. RNA Extraction, Reverse Transcription and qPCR

Total RNA was extracted from cell lines and primary thyroid tumors using Trizol-Reagent (Thermo Fisher Scientific), and mirVana miRNA Isolation Kit (Invitrogen, Carlsbad, CA, USA), respectively. RNA was reversely transcribed using High Capacity cDNA Reverse Transcription Kit (Thermo Fisher Scientific). qPCR was performed in QuantStudio 7 Flex Real-Time PCR System using SYBR Green (Thermo Fisher Scientific). Relative expression levels of *PLEKHS1* mRNA were calculated based on the ΔCT values and normalized to human *β2-M* expression [9]. The following primers were used in this study for *PLEKHS1*: 5′-GGT CCA GAC CAG GTC TCT GGA-3′ (Forward) and 5′-CCC CAT CCT GGG TCT CTG GA-3′ (Reverse). PCR annealing temperature was 60 °C, and products were 144 bp long fragments.

### 4.5. Plasmid Transfection

PLEKHS1 expression plasmids were purchased from AddGene (Watertown, MA, USA). TC cells grown in 6-well plates were transfected with empty control or PLEKHS1 expression vectors by using Lipofectamine3000 (Thermo Fisher Scientific) according to the protocol provided.

### 4.6. Western Blot Analysis

Total cellular proteins were extracted from cell lines and PTC tumors using RIPA Lysis Buffer (Thermo Fisher Scientific) with 1% Phenylmethanesulfonyl fluoride (Sigma-Aldrich, Darmstadt, Germany) with or without Protease Inhibitor Cocktail (Sigma-Aldrich, Darmstadt, Germany). For the analysis of phosphorylated AKT, cells were washed once with sodium orthovanadate-containing buffers to inhibit phosphatases prior to RIPA buffer lysis. Thirty micrograms of proteins were separated in Mini-PROTEAN TGX Gels (Bio-Rad Laboratories, Hercules, CA) and transferred to PVDF membranes using Trans-Blot Turbo Transfer Pack (Bio-Rad). Membranes were blocked with 5% non-fat milk diluted in TBST, and then incubated with primary antibodies and secondary antibodies before being imaged with Clarity Max Western ECL Substrate (Bio-Rad, 1705062) and ChemiDoc MP Imaging System (Bio-Rad). The following primary antibodies were used: anti-PLEKHS1 (Novus Biologicals, H00079949-M07) at dilution 1:500, anti-AKT (Cell Signaling Technology, Boston, MA, USA, 9272) and phosphorylated-AKT (Cell Signaling Technology, 4051) at dilution 1:1000, and anti-*β*-Actin (Santa Cruz, sc-47778, Dallas, TX, USA) at dilution 1:50,000. Secondary antibodies include Goat Anti-Mouse IgG (H + L)-HRP Conjugate (Bio-Rad, 170-6516) and Goat Anti-Rabbit IgG (H + L)-HRP Conjugate (Bio-Rad, 170–6515).

### 4.7. Pyrosequencing for DNA Methylation Analyses

Genomic DNA was bisulfited-converted using EpiTect Bisulfite Kits (Qiagen, Hilden, Germany) followed by PCR amplification using PyroMark PCR Kit (Qiagen) with PLEKHS1 promotor specific primers. The reverse primer was biotin-labeled to purify the PCR product by binding to streptavidin-coated Sepharose beads (GE Healthcare, UK). PCR fragments were bound to Sepharose beads followed by repeated washing and denaturation. Beads were then mixed with sequencing primer and annealed of sequencing primer to DNA. The plate was then transferred to a PyroMark Q96 (Qiagen) to perform sequencing according to the manufacturer’s instructions. The primers for PCR and sequencing were: PLEKHS1 PCR primers: 5′-TTT TTA GGA AGA TAT TGG TTA AGA TAT GG-3′ (forward) and 5′-Biotin-TCC TAC CAA ACT TTA AAC CAT AAT CAC AAT-3′ (reverse); PLEKHS1 sequencing primer: 5′- GGG ATT TTT TTT TAA TGG TAG T-3′.

### 4.8. Transwell Assays for Cell Migration and Invasion

A transwell assay system was used to determine the migration and invasion capacities of TC cells as described [9,31]. MDA-T32, MDA-T41 and U-hth-104 cells transfected with control or PLEKHS1 expression vectors (1 × 10^5^) were seeded into the upper chamber. The low chamber contained RPMI-1640 medium with 20% FBS. The migrated cells were stained with crystal violet, counted and photographed 24 h later. For invasion assay, 50 μL matrigel (Corning Life Sciences, Flintshire, UK) was first loaded into the bottom of the upper chamber followed by the identical procedure as described above.

### 4.9. Statistical Analyses

All statistical analyses were performed using IBM SPSS Statistics version 24 (IBM, Armonk, NY, USA). Student’s *t*-test or Mann–Whitney U-test was used for comparison of differences between groups. Spearman’s or Pearson’s correlation coefficient was applied to determine correlation coefficient r. Survival analyses were performed with log-rank test and univariate Cox regression. OS and DFS were visualized with Kaplan–Meier plots. *PLEKHS1* mRNA levels were classified into two groups based on the median separation. Multivariate analysis was performed with a Cox regression model based on variables that were significant in univariate Cox regression. *p*-values below 0.05 were considered as statistically significant.

## 5. Conclusions

The results presented herein reveal that PLEKHS1 is over-expressed in thyroid carcinomas including PTCs and ATCs, and its higher expression is associated with lethal ATCs as well as metastatic PTCs and shorter patient survival. Mechanistically, unknown oncogenic events may drive the aberrant demethylation of the *PLEKHS1* locus, thereby enhancing its expression. Moreover, PLEKHS1 may contribute to the hyperactivity of AKT, and consequently promotes aggressive PTCs. Our findings are thus of both biological and clinical importance.

## Figures and Tables

**Table 1:** Clinical characteristics and statistical comparison for the 93 PTC cases.

Parameters *n* = (No of Informative Cases)	Observations	*PLEKHS1* mRNA	*p*-Value
		Median (Min–Max)	
Age at diagnosis (*n* = 93)			0.365
Median (Min–Max) years	51 (15–97)		
<55	*n* = 55	1.43 (0.21–139.30)	
≥55	*n* = 38	1.67 (0.37–811.88)	
Gender (*n* = 93)			0.980
Female	*n* = 67	1.43 (0.21–811.88)	
Male	*n* = 26	1.77 (0.21–421.76)	
Tumor size (*n* = 88)			0.244
≤2cm (*n* = 40)	*n* = 40	1.06 (0.21–11.30)	
2–4cm (*n* = 29)	*n* = 29	2.09 (0.21–811.88)	
>4cm (*n* = 19)	*n* = 19	1.67 (0.45–139.30)	
Lymph node metastases (*n* = 93)			0.026
No	*n* = 43	1.17 (0.21–421.76)	
Yes	*n* = 50	1.70 (0.21–811.88)	
Distant metastases (*n* = 93)			0.011
No	*n* = 81	1.28 (0.21–421.76)	
Yes	*n* = 12	3.43 (0.54–811.88)	
Extrathyroidal extension (*n* = 81)			0.833
No	*n* = 47	1.26 (0.21–97.07)	
Yes	*n* = 34	1.36 (0.22–811.88)	
*TERT* hotspot promoter mutation (*n* = 93)			0.281
Wild-type	*n* = 64	1.35 (0.21–421.76)	
Mutation C228T/C250T	*n* = 24/5	1.86 (0.37–811.88)	
*TERT* mRNA expression (*n* = 93)			0.603
No *TERT* mRNA	*n* = 40	1.46 (0.21–139.30)	
*TERT* mRNA expressed	*n* = 53	1.59 (0.37–811.88)	
Overall survival (*n* = 93)			<0.001
Alive	*n* = 32	1.17 (0.21–139.30)	95% CI = 1.002–1.007
Dead	*n* = 61	2.17 (0.37–811.88)	*HR* = 1.005
Follow-up:median (min–max) years	14.8 (0.2–26.5)		
Disease-free survival (*n* = 93)			0.004
No evidence of disease	*n* = 67	1.26 (0.21–421.76)	95% CI = 1.001–1.007
Relapsed/progression	*n* = 26	2.09 (0.34–811.88)	*HR* = 1.004
Follow-up: Median (Min–Max) years	13.5 (0.1–26.5)		

HR: hazard ratio, 95% CI: 95% confidence interval. Mann–Whitney U-test or Kruskal–Wallis Test was used for comparison between groups. Univariate Cox-regression was used for survival analysis, *PLEKHS1* mRNA as. a continuous variable. *n*: number.

**Table 2:** Clinical characteristics and statistical comparison for the 18 ATC cases.

Parameters (Number of Informative Cases)	Observations	*PLEKHS1* mRNA	*p*-Value
		Median (Min–Max)	
Age at diagnosis (*n* = 18)			
Median (Min–Max) years	77.5 (54–91)		0.101
<55 years	*n* = 1	1.03 (1.03–1.03)	
≥55 years	*n* = 17	4.00 (1.80–85.57)	
Gender (*n* = 18)			0.214
Female	*n* = 10	4.68 (2.92–85.57)	
Male	*n* = 8	2.76 (1.03–18.91)	
Tumor size (*n* = 18)			0.251
≤2 cm	*n* = 1	1.03 (1.03–1.03)	
2–4 cm	*n* = 2	44.04 (2.52–85.57)	
>4 cm	*n* = 15	4.00 (1.80–18.91)	
*TERT* promoter mutation (*n* = 18)			0.722
Wild-type	*n* = 8	4.26 (1.03–85.57)	
Mutation (C228T/C250T)	*n* = 7/3	3.67 (1.80–18.91)	
TERT mRNA expression (*n* = 18)			0.888
No TERT mRNA	*n* = 2	10.02 (2.33–17.71)	
TERT mRNA expressed	*n* = 16	3.67 (1.03–85.57)	
Survival (*n* = 17)			0.54
Alive	*n* = 1	7.91 (7.91–7.91)	
Dead	*n* = 16	3.25 (1.03–85.57)	
Follow-up: Median (Min–Max) months	3 (0–190)		

*n*: numbers. HR: hazard ratio, 95% CI: 95% confidence interval. Mann-Whitney U-test or Kruskal-Wallis Test was used for comparison between groups.

## Supplementary Materials

The following are available online at https://www.mdpi.com/2072-6694/12/8/2133/s1, Table S1: Univariate and multivariate Cox regression analyses of overall survival in 93 patients with papillary thyroid carcinoma, Table S2: Univariate and multivariate Cox regression analyses of disease-free survival in 93 patients with PTC, Table S3: Clinical characteristics and statistical comparison for the 393 PTC cases in TCGA.
