# Supplementary material for: PLEKHS1 Over-Expression is Associated with Metastases and Poor Outcomes in Papillary Thyroid Carcinoma
Source: Cancers (Basel). 2020 Jul 31;12(8):2133. doi: 10.3390/cancers12082133 (PMC7465481; doi:10.3390/cancers12082133)

cancers

Article

PLEKHS1 Over-Expression Is Associated with Metastases and Poor Outcomes in Papillary Thyroid Carcinoma

Xiangling Xing, Ninni Mu, Xiaotian Yuan, Na Wang, C. Christofer Juhlin, Klas Strååt, Catharina Larsson and Dawei Xu

Supplementary Materials

M1: Checked without problems.
M2: It is wrong in the online system. The corresponding authors should be Xiaotian Yuan and Catharina Larsson.
M3: Confirmed.
M4: No.
M5 and M6: Bold is necessary.
M7: No.
M8: The correction was made for figure 3.
M9, M14, M16, M17, M19 and M21: Appropriate information was added.
M10: The correction was made for figure 4.
M11: Confirmed.
M12: The correction was made for figure 5.
M15: The correction was made for figure 6.
M18: The information was added.
M22: Agree.
M23: Confirmed.
M24 and M25: Confirmed.

Table S1. Univariate and multivariate Cox regression analyses of overall survival in 93 patients with papillary thyroid carcinoma.

Parameters	Univariate analyses				Multivariate analyses			
	**B coefficient**	**HR**	**95% CI**	***p*-value**	**B coefficient**	**HR**	**95% CI**	***p*-value**
***PLEKHS1* mRNA**								
Low expression (n = 46)	Ref	Ref	Ref		Ref	Ref	Ref	
High expression (n = 47)	0.939	2.556	1.209–5.408	**0.014**	0.988	2.685	1.139–6.332	**0.024**
***TERT* promoter mutation**								
Wild-type (n = 64)	Ref	Ref	Ref		Ref	Ref	Ref	
Mutation C228T / C250T (n = 24/5)	2.145	8.542	3.907–18.674	**< 0.001**	-0.664	0.515	0.135–1.963	0.331
***TERT* mRNA expression**								
No *TERT* mRNA (n = 40)	Ref	Ref	Ref		Ref	Ref	Ref	
TERT mRNA expressed (n = 53)	2.619	13.728	3.278–57.499	**< 0.001**	1.136	3.114	0.580–16.726	0.185
**Age**								
<55 years (n = 55)	Ref	Ref	Ref		Ref	Ref	Ref	
≥55 years (n = 38)	2.823	16.830	5.811–48.740	**< 0.001**	3.014	20.375	4.329–95.905	**<0.001**
**Gender**								
Female (n = 67)	Ref	Ref	Ref		Ref	Ref	Ref	
Male (n = 26)	0.641	1.899	0.934–3.859	0.076	-0.586	0.557	0.226–1.372	0.203
**Tumor size**				**0.001**				**0.023**
≤2 cm (n = 40)	Ref	Ref	Ref		Ref	Ref	Ref	
2–4 cm (n = 29)	1.024	2.786	1.030–7.537	**0.044**	0.301	1.351	0.378–4.824	0.643
>4 cm (n = 19)	1.864	6.451	2.413–17.246	**< 0.001**	1.376	3.961	1.170–13.404	**0.027**

Table S2. Univariate and multivariate Cox regression analyses of disease-free survival in 93 patients with PTC.

Parameters	Univariate analyses				Multivariate analyses			
	**B coefficient**	**HR**	**95% CI**	***p*-value**	**B coefficient**	**HR**	**95% CI**	***p*-value**
***PLEKHS1* mRNA**								
Low expression (n = 46)	Ref	Ref	Ref		Ref	Ref	Ref	
High expression (n = 47)	0.813	2.255	1.004–5.067	**0.049**	0.327	1.387	0.569–3.384	0.472
***TERT* promoter mutation**								
Wild-type (n = 64)	Ref	Ref	Ref		Ref	Ref	Ref	
Mutation C228T / C250T (n = 24 / 5)	1.927	6.872	3.029–15.587	**<0.001**	0.757	2.132	0.222–20.463	0.512
***TERT* mRNA expression**								
No *TERT* mRNA (n = 40)	Ref	Ref	Ref		Ref	Ref	Ref	
TERT mRNA expressed (n = 53)	1.181	3.257	1.305–8.133	**0.011**	0.177	1.193	0.276–5.158	0.813
**Age**								
< 55 years (n = 55)	Ref	Ref	Ref		Ref	Ref	Ref	
≥ 55 years (n = 38)	1.468	4.34	1.913–9.847	**<0.001**	0.221	1.248	0.149–10.418	0.838
**Gender**								
Female (n = 67)	Ref	Ref	Ref		Ref	Ref	Ref	
Male (n = 26)	1.056	2.874	1.323–6.241	**0.008**	0.407	1.503	0.610–3.701	0.376
**Tumor size**				**0.002**				0.055
≤2 cm (n = 40)	Ref	Ref	Ref		Ref	Ref	Ref	
2–4 cm (n = 29)	1.850	6.358	1.792–22.562	**0.004**	1.307	3.694	0.895–15.245	0.071
>4 cm (n = 19)	2.411	11.141	3–41.377	**<0.001**	1.76	5.812	1.381–24.456	**0.016**

Table S3. Clinical characteristics and statistical comparison for the 393 PTC cases in TCGA.

Parameters (Number of Informative Cases)	Observations	*PLEKHS1* mRNA Median (Min–Max)	*p*-value
**Age at diagnosis (n = 393)**			
Median (Min–Max) years	46 (15–89)		0.312
< 55 years	n = 218	2.89 (0–1326.92)	
≥ 55 years	n = 175	2.10 (0–891.32)	
**Gender (n = 393)**			0.895
Female	n = 285	2.33 (0–1326.92)	
Male	n = 108	2.65 (0–891.31)	
**Disease stage (n = 392)**			0.128
Stage I + II	n = 251	2.20 (0–1326.92)	
Stage III + IV	n = 141	3.15 (0–573.90)	
**Lymph node metastases (n = 367)**			**0.002**
No	n = 161	1.91 (0–891.32)	
Yes	n = 206	3.24 (0–1326.92)	
**Distant metastases (n = 249)**			0.758
No	n = 245	2.88 (0–1326.92)	
Yes	n = 4	4.37 (0–7.888)	
***TERT* promoter mutation (n = 312)**			0.545
Wild-type	n = 281	2.55 (0–1326.92)	
Mutation (C228T/C250T)	n = 23/8	2.79 (0–573.91)	
**TERT mRNA expression (n = 393)**			**< 0.0001**
No TERT mRNA	n = 275	2.09 (0–1326.92)	
TERT mRNA expressed	n = 118	4.03 (0–573.91)	
**Overall survival (n = 393)**			0.290
Alive	n = 378	2.58 (0–1326.92)	95% CI = 0.999–1.004
Dead	n = 15	1.65 (0–573.91)	HR = 1.001
Follow-up: Median (Min–Max)months	31.47 (0–178)		
**Disease-free survival (n = 380)**			0.548
No evidence of disease	n = 340	2.63 (0–1326.92)	95% CI = 0.985–1.008
Relapsed/progression	n = 40	2.00 (0–144.60)	HR = 0.997
Follow-up:Median (Min–Max) months	31 (0–178)		

n: numbers; HR: hazard ratio; 95% CI: 95% confidence interval. Univariate Cox-regression was uesd for survival analysis, *PLEKHS1* mRNA as a continuous variable. Mann-Whitney U-test were used for comparison between groups.

Original immunoblotting images presented in the paper

Figure 2A

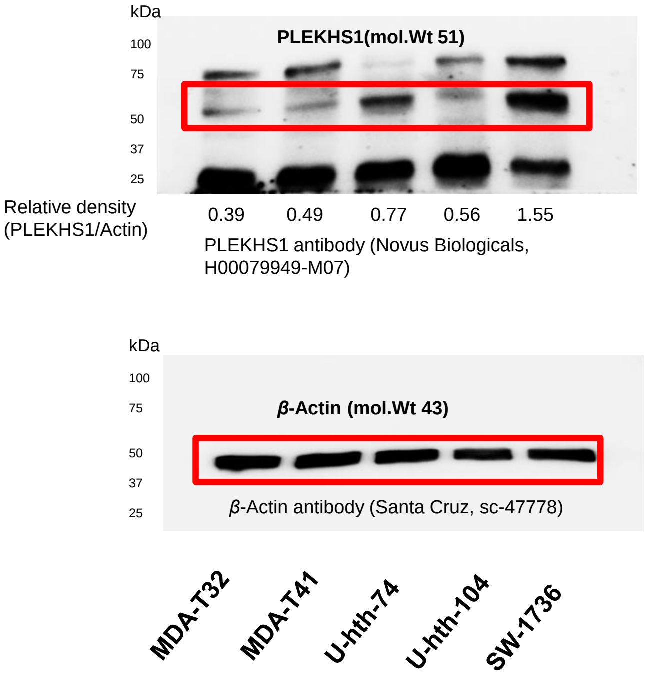

Figure 2D

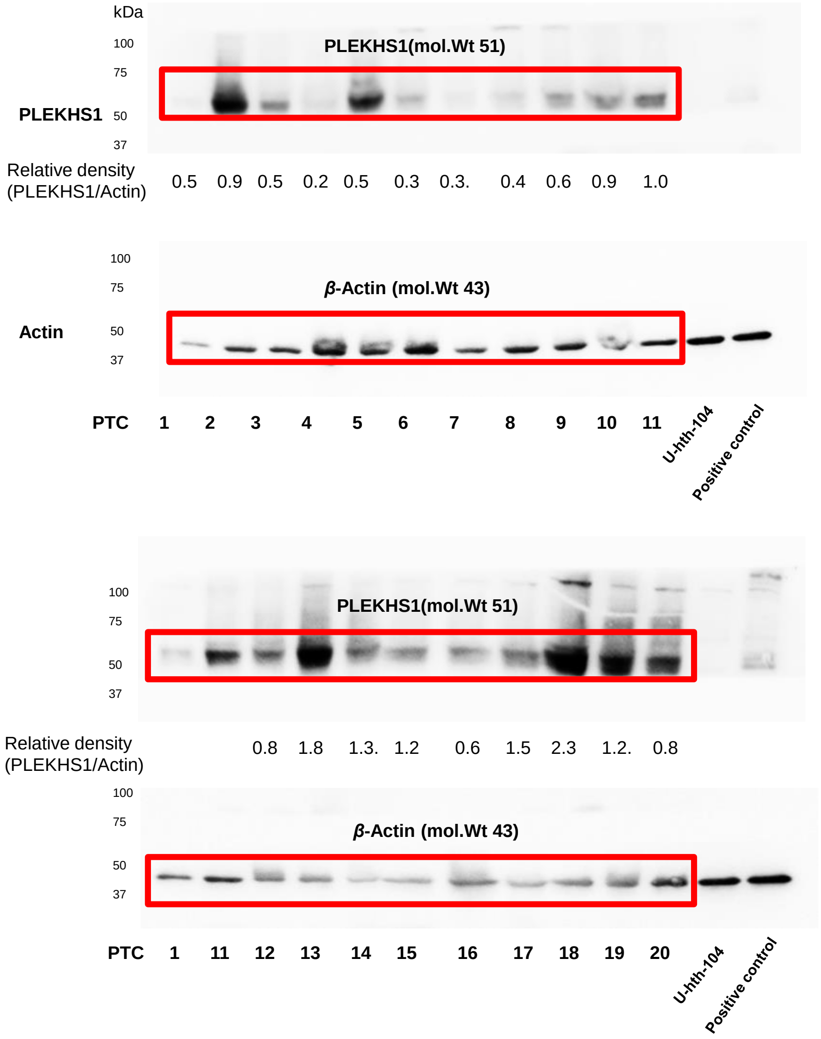

Figure 4A

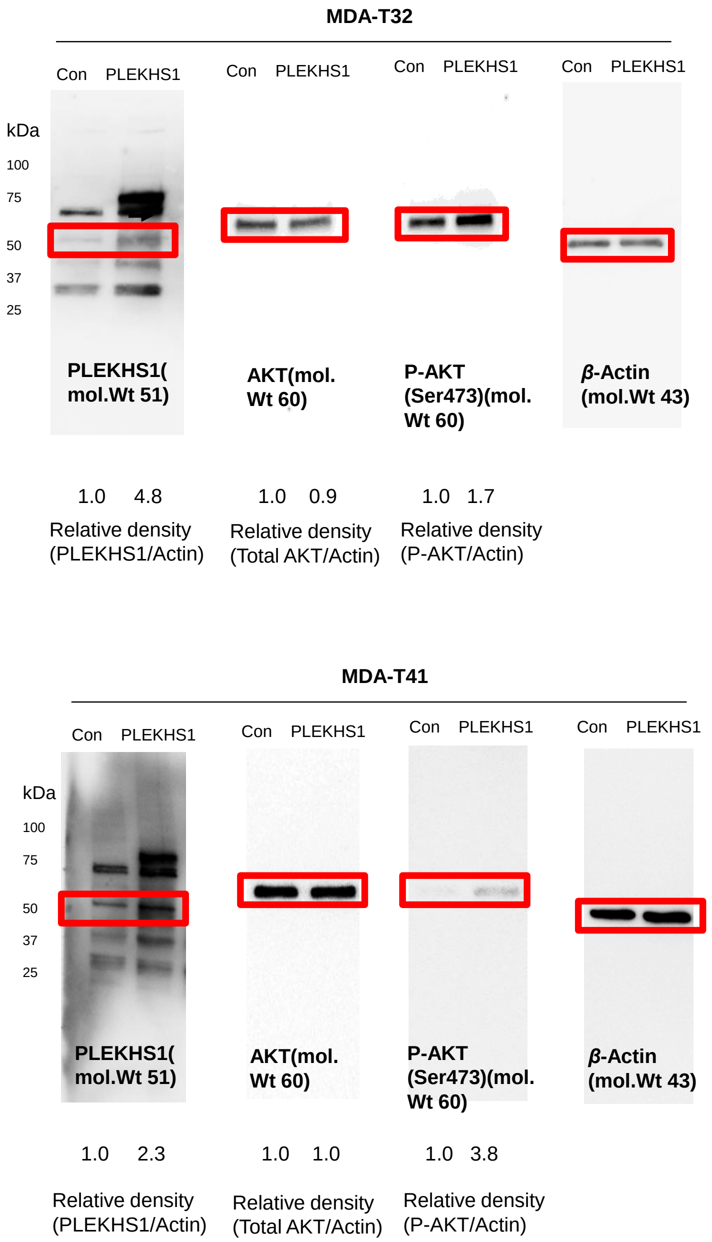

Figure 4A

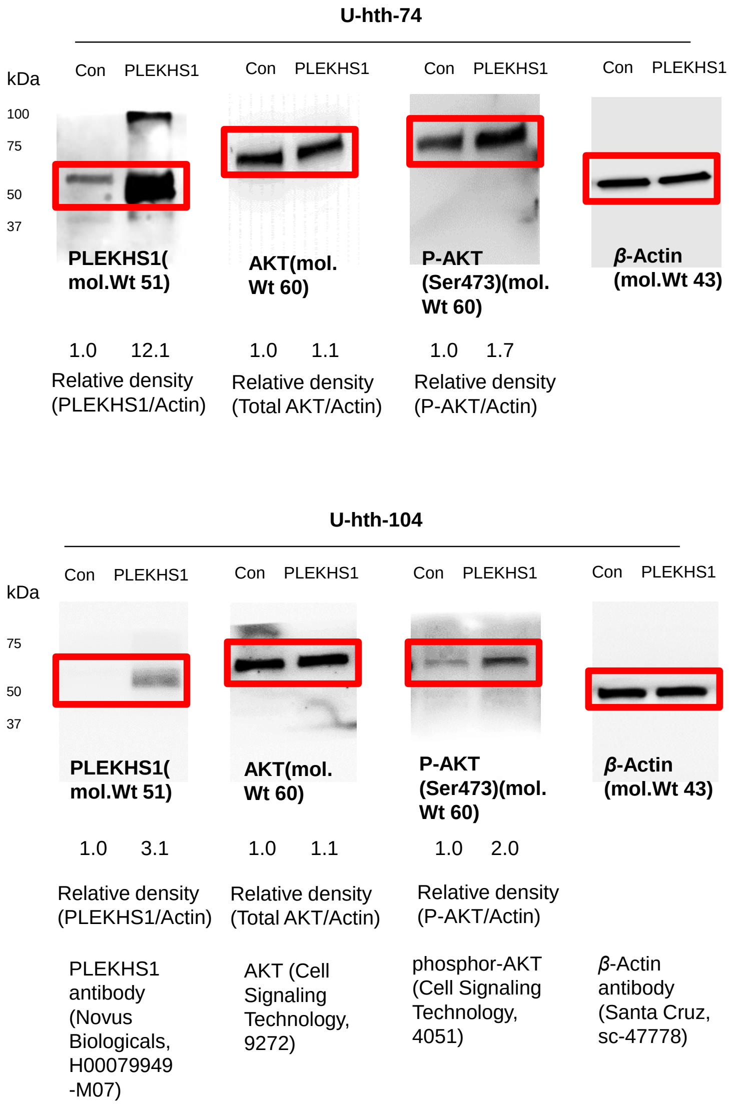

Supplement: Supplementary file 1 [file cancers-12-02133-s001.pdf]
